# Prenatal Diagnosis of Acrania in One Twin of a Dichorionic Diamniotic Pregnancy: A Case Report on Management and Perinatal Outcome

**DOI:** 10.3390/reports8020075

**Published:** 2025-05-22

**Authors:** Agnieszka Żalińska, Weronika Marcinkowska, Filip Gągorowski, Łukasz Sokołowski, Michał Krekora, Przemysław Oszukowski, Agnieszka Pięta-Dolińska

**Affiliations:** 1 Department of Obstetrics and Perinatology, Chair of Obstetrics, Gynecology and Oncological Gynecology, Medical University of Lodz, 90-419 Lodz, Poland; oszukowskip@gmail.com (P.O.); pietadol@gmail.com (A.P.-D.); 2 Department of Gynecology, Reproduction and Fetal Therapy and Infertility Diagnostics and Treatment, Polish Mother’s Memorial Hospital Research Institute, 93-338 Lodz, Poland; 3 Faculty of Medicine, Medical University of Lodz, 90-419 Lodz, Poland; w.marcinkowska88@gmail.com (W.M.); filip.g.fgg@gmail.com (F.G.); 4 Department of Obstetrics and Gynecology, Polish Mother’s Memorial Hospital Research Institute, 93-338 Lodz, Poland; l.sokolowski90@gmail.com (Ł.S.); krekoram@poczta.onet.pl (M.K.); 5 Department of Gynecology and Obstetrics, Medical University of Lodz, 90-419 Lodz, Poland; 6 Labour Department, Polish Mother’s Memorial Hospital Research Institute, 93-338 Lodz, Poland

**Keywords:** acrania, neural tube defects, dichorionic diamniotic twin pregnancy

## Abstract

**Background and Clinical Significance**: Twin pregnancies are associated with an increased risk of congenital malformations. One of them is rare but lethal—acrania—which belongs to the group of neural tube defects. The pathogenesis of acrania is not fully understood. It is presumed that the underlying mechanism of its development is a disorder of migration of mesenchymal tissue. The presence of an acrania in one of the twins may lead to complications such as polyhydramnios, preterm labor, or, in severe cases, an intrauterine death in one or both twins. **Case Presentation**: A 30-year-old woman (G4P4) was admitted to the Labor Department of a tertiary hospital in 30+3 weeks due to preterm labor. The patient was in a dichorionic diamniotic twin pregnancy with a single lethal fetal anomaly and severe polyhydramnios of a second twin. Hence, the caesarean section was immediately performed. Both twins were admitted to the Neonatology Department. The healthy neonate was hospitalized and discharged after 42 days in good condition. Palliative care for the twin with acrania was provided. **Conclusions**: Early detection of acrania in twin pregnancies is critical. It allows the implementation of appropriate management and targeted counseling, thereby minimizing the risk of complications both for unaffected twins and the mothers. Our case is a good model of action where a twin pregnancy with a diagnosed lethal defect in an ambulatory setting was managed, providing holistic specialized care.

## 1. Introduction and Clinical Significance

Twin pregnancies complicated by a major fetal anomaly in one of the fetuses present unique diagnostic, ethical, and clinical challenges [1]. Acrania is a rare, lethal defect characterized by abnormal development of cranial bone structures. It represents the first stage of the acrania–exencephaly–anencephaly sequence (AEAS), a complex defect with a frequency of 3.68 to 5.4 per 10,000 live births [2,3]. Acrania more often affects Caucasian and Hispanic women and female fetuses [4]. The development of acrania is thought to result from abnormal migration of mesenchymal tissue, which usually covers the cerebral hemispheres and contributes to the formation of the membranous neurocranium [5] During the fourth week of embryonic development, this mesenchymal layer—derived from paraxial mesoderm and neural crest cells—migrates beneath the surface ectoderm to envelop the developing brain, giving rise to the flat bones of the cranial vault. Failure of this process leads to the absence of calvarial bones, leaving the brain exposed and vulnerable to secondary degeneration [6]. While the exact etiology is multifactorial—incorporating genetic, nutritional, and environmental factors—folic acid deficiency remains a well-established contributor to neural tube defects (NTDs) [4]. Ultrasound (US) is the primary method of acrania diagnosis, particularly after the first trimester routine scan, which is performed between 11 + 0 and 13 + 6 weeks of pregnancy when a skull ossification should occur [7,8]. Fetal magnetic resonance imaging (MRI) can provide adjunctive value in complex or equivocal cases [1,9]. Beyond diagnostic and developmental considerations, the diagnosis of a lethal anomaly in one fetus of a twin pregnancy presents significant ethical and psychological challenges. Parents must weigh decisions about pregnancy continuation, fetal interventions, and the impact on the co-twin. Emotional distress and psychological trauma are common in such contexts and require dedicated psychosocial support [10]. In this report, we present a case of dichorionic diamniotic twin pregnancy complicated by acrania in one fetus, diagnosed during the first-trimester US examination. The objective of this case is to highlight the importance of early imaging, the value of sequential prenatal monitoring, and the necessity of individualized, holistic care in managing such ethically and emotionally complex pregnancies.

## 2. Case Presentation

A 30-year-old woman, gravida 4, para 4 (G4P4), in a dichorionic diamniotic twin pregnancy with a single lethal fetal anomaly detected during the first trimester, was admitted to the hospital. Prenatal US revealed an average anatomy of one of the twins; however, the second twin was diagnosed with acrania. The patient previously had three vaginal deliveries without any complications. In addition, she had no significant family, medical or surgical history of congenital anomalies. There was also no history of use of teratogenic drugs during pregnancy. Folic acid supplementation was started when the pregnancy was confirmed, at 6 weeks. No supplementation was used during the preconception period. However, there was considerable doubt related to the regularity of the patient’s folic acid intake.

During the second and third trimesters of pregnancy, the patient remained under irregular obstetric control. The patient rejected repeated suggestions for hospitalization to expand the diagnosis. A routine echocardiography examination was performed, which revealed no abnormalities. General cooperation with the patient was difficult, and her many refusals interfered with the proper diagnostic and therapeutic process. Despite the indications related to increasing polyhydramnios, she refused to consent to the amnioreduction procedure.

**Figure 1:**
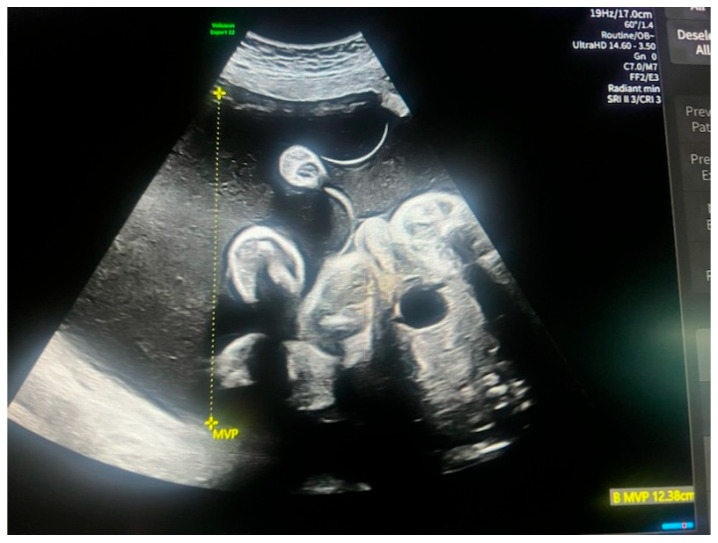
The measurement of maximal vertical pocket in dichorionic diamniotic pregnancy (own material).

The patient was admitted at 32 + 3 weeks to the Labor Department of Polish Mother’s Memorial Hospital Research Institute. Obstetric examination revealed advanced labor and premature preterm rupture of membrane (pPROM). A US scan showed two twins in a cephalic position. In twin 2, severe polyhydramnios was detected (maximal volume pocket (MVP) was estimated at 15 cm) (Figure 1). Due to the advanced polyhydramnios, and the high risk of altering the presentation of twin 2, it was decided to perform a cesarean section with the consent of the patient.

**Figure 2:**
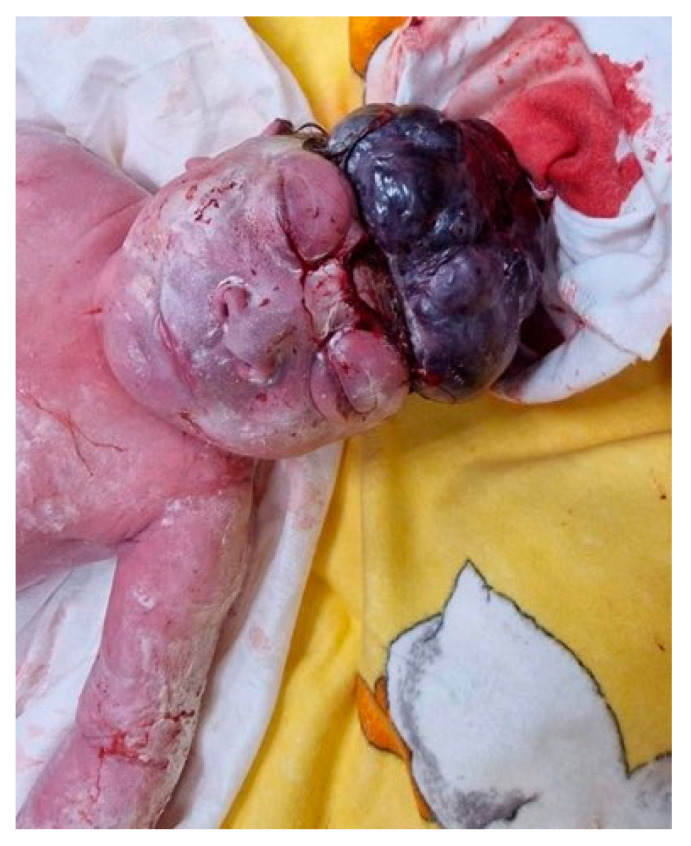
The newborn with acrania (own material).

A healthy male twin weighing 2250 g was delivered with Apgar scores of 6/6/6/6. He was admitted to the Department of Neonatology due to prematurity. A live female twin 1 with an acrania weighing 1500 g was delivered with Apgar’s score of 2/2/3/3 and was admitted to the Neonatology Clinic with a plan of conservative management for palliative care (Figure 2). A few hours later, twin 1 passed away due to cardiorespiratory arrest. In contrast, twin 2 was discharged from the hospital in good condition after 42 days. As well, the hospitalization showed no abnormalities after ophthalmology, cardiology, and neurology consultations. The mother was discharged 6 days after delivery in good somatic and psychiatric condition.

## 3. Discussion

The onset of acrania’s development occurs 10 to 20 days after ovulation when the anterior neural groove fails to close [11]. The failure of mesenchymal tissue to move into the region of the cerebral hemispheres results in the absence of cranial bone, and the only protection for the brain remains the excess ectoderm, forming a thin amniotic membrane. The lack of brain tissue protection is associated with exposure to the damaging effects of amniotic fluid through increased urea concentration and elevated risk of mechanical injury due to friction against the uterine wall and placenta. Consequently, it leads to gradual destruction and degeneration of brain tissue [5,11].

Similarly to other NTDs, the etiology of acrania is multifactorial due to complex interactions between genes and the environment. Among genetic factors, key roles are played by genes involved in folate metabolism, including the methylenetetrahydrofolate reductase gene (MTHFR) and Van Gogh-like planar cell polarity protein 1 (VANGL1). Additionally, NTDs can be associated with chromosomal abnormalities and amniotic membrane disorders [4]. In contrast, the most commonly mentioned environmental factors are smoking, maternal nutritional status, uncontrolled gestational diabetes, hyperthermia, emotional stress, and air pollution [4,12,13]. In a meta-analysis conducted by Vena F. et al., the risk of NTDs according to pre-pregnancy body mass index was evaluated [14]. Obtained results highlight that obese pregnant women have a significantly higher risk of developing fetuses with NTDs compared to normal-weight women (OR 1.62 95% CI 1.32–1.99, *p* < 0.0001). However, there was no difference in NTD risk in underweight and overweight mothers [14]. Importantly, medications taken during pregnancy are also of critical importance. Daham N. et al. described a case of fetal acrania in a pregnant woman taking adalimumab, which is a human recombinant IgG1 monoclonal antibody [15]. Moreover, Gorgal R. et al. analyzed the etiology of acrania in 14 fetuses diagnosed in the first or early second trimester of pregnancy [4]. Among the women who participated in the study, one had epilepsy and was taking anticonvulsants, while another suffered from uncontrolled diabetes. Additionally, most of them did not take folic acid at the time of conception. Also, in three cases, cytogenetic analysis revealed chromosomal abnormalities in the form of partial deletion of chromosome 18 and trisomy 18 [4]. One of the most well-established and modifiable risk factors for NTDs is folic acid deficiency during the periconceptional period. Research indicates that adequate supplementation can reduce the risk of NTDs by over 70% [16,17,18]. The biological basis lies in folate’s critical function in DNA synthesis, repair, and methylation, which are processes fundamental for proper embryonic cell division and differentiation. Folate deficiency can lead to mutations or abnormal gene methylation in DNA repair pathways, promoting genomic instability and altering gene expression, which can disrupt the balance between apoptosis and proliferation during neural tube development [19]. The neural plate completes its formation and closure at a very early stage of pregnancy, typically around 28 days after fertilization [20]. This means that the critical period for folic acid supplementation begins at least one month prior to conception and continues through the first 2 to 3 months of pregnancy [21]. Current guidelines recommend daily folic acid supplementation (400–800 μg) for all women who are planning a pregnancy or could become pregnant [20]. Ideally, supplementation should begin at least one month before the intended conception and be continued throughout the first trimester [22]. In high-risk populations—such as women with a previous pregnancy affected by an NTD—a higher dose of up to 5 mg per day may be recommended [23,24]. In the context of our case, uncertainty regarding the patient’s regular intake of folic acid during the periconceptional period remains a relevant factor when considering the etiology of the defect. This highlights the ongoing need for education aimed at promoting adequate folic acid supplementation among women of reproductive age. Technical standards for the US in diagnosing acrania are described in the literature. Ştefănescu BI. et al. recommended examining more than the midsagittal, axial, and coronal planes to better identify frontal bone ossification [25]; an important sonographic feature is the absence of echogenic calvarium and brain landmarks appropriate for gestational age. Moreover, Szkodziak P. et al. described the “beret” sign as the appearance of a thin, inert, rippled membrane surrounding the brain structures and an anechoic space that corresponds to the cerebrospinal fluid [5].

In addition, there is a characteristic image of a typical bilobed or “Mickey Mouse” shaped head, which corresponds to the cerebellar hemispheres being exposed in the frontal plane [11].

During the second trimester, a significant amount of brain tissue atrophies, especially above the level of the fetal orbits, which is referred to as “frog face” or “frog eyes” [5]. Along with brain atrophy and the development of AEAS, significantly increased echogenicity of the amniotic fluid can also be observed (89% of cases) [2]. Lastly, in the second half of the second trimester and the third trimester of pregnancy, polyhydramnios occur in about 50% of cases [11].

The presented case reveals that the diagnosis of a defect in one fetus, even of a lethal character, allows the pregnancy to progress and preserve the other fetus. In such circumstances, sequential US monitoring is crucial to constantly monitor both fetuses’ conditions. Additionally, echocardiography should be conducted. It is essential not only for the detection of heart structural defects that may coexist with NTDs, but also for detecting possible cardiac issues in the second fetus. It is noteworthy that the presence of concomitant congenital heart defects is associated with increased mortality, premature deliveries, and decreased birth weight [26]. Symptoms indicative of hemodynamic overload in an unaffected fetus may be the first sign of fetal distress. However, in the case of our patient, echocardiography did not show any abnormalities.

Twin pregnancies are associated with an elevated risk of congenital malformations compared to singleton pregnancies [1]. Research conducted by Syngelaki A. et al. based on an ultrasound analysis of 6366 twin pregnancies at 11–13 weeks of gestation showed that the overall incidence of fetal abnormalities was increased in monochorionic twin pregnancies compared to dichorionic twin pregnancies [27]. NTDs occur in twins at a prevalence of 2.3/1000, with the majority of cases involving twin pregnancies discordant for fetal anomaly [1]. A defect in one twin can significantly affect the other twin, causing difficulties in the decision-making for both parents and doctors [28]. A procedure to minimize the risks for the developing pregnancy and the intact twin is selective termination of the affected twin. The procedure is recommended to be performed before 18 weeks gestation, and the decision on the technique depends on chorionicity. Umbilical cord occlusion is the preferred method for monochorionic pregnancies, while for dichorionic pregnancies, an intracardiac injection of potassium chloride or lignocaine is performed [1]. Kristensen SE. et al. evaluated the risk of pregnancy complications [29]. The study included 9735 twin pregnancies of dichorionic twins, 172 of which were reduced between 11 and 23 weeks of pregnancy by transabdominal injection of potassium chloride. Twins that were not reduced were delivered significantly earlier than reduced twins (*p* < 0.01), by an average of 15 days for twins reduced before 14 weeks and 10 days for twins reduced from 14 weeks [29]. In our case, the procedure was excluded due to Polish legal regulations. The selective termination of a fetus with a lethal anomaly involves significant bioethical considerations. This situation requires careful reflection on various ethical dilemmas, with full respect for the beliefs, values, and rights of the mother. One of the primary ethical tensions arises from the conflict between pro-choice and pro-life perspectives, particularly regarding a woman’s right to make autonomous decisions about her own body. In addressing this dilemma, it is essential that selective termination is based on clear medical indications confirmed through prenatal diagnostic procedures. However, the reliability of such diagnoses may occasionally be questioned, adding further complexity to the decision-making process. In twin pregnancies, the well-being of both fetuses must be taken into account. A fetus diagnosed with a lethal anomaly is likely to pass away after birth. During pregnancy, the affected twin may complicate the course and impair the healthy twin’s prenatal development. This raises an ethical question: should the compromised condition of one twin be allowed to negatively affect the growth and survival of the other, and could intervention improve the prognosis for the healthy twin. Nevertheless, it is essential to recognize that the selective termination carries inherent medical risks, including the possibility of miscarriage or preterm labor. The ethical issues surrounding this procedure are highly controversial and tend to divide public opinion into supporters and opponents. Ultimately, for healthcare professionals, the most important principle is to respect the beliefs, social values, and informed decisions of the woman involved [30,31]. If the pregnancy continues, the possibility of intrauterine intervention should be highlighted. In cases of anatomical defects such as acrania, there is an increased risk of polyhydramnios, which is typically classified into three severity levels based on the amniotic fluid index (AFI): mild (25.0–29.9 cm), moderate (30.0–34.9 cm), and severe (≥35.0 cm) [32]. Polyhydramnios is associated with adverse obstetric outcomes, including maternal dyspnea, preterm delivery, premature rupture of the amniotic membranes, umbilical cord prolapsed, perinatal death, and postpartum hemorrhage [33,34]. However, a retrospective cohort study demonstrated a significantly higher rate of cesarean sections in the polyhydramnios group compared to controls [32]. That is why an amnioreduction procedure is suggested. The primary rationale is to restore a normal amniotic fluid volume in order to prolong the pregnancy by reducing the risk of preterm labor and spontaneous rupture of membranes. Amnioreduction is also offered to patients experiencing significant clinical symptoms, such as dyspnea, abdominal and respiratory discomfort, and other issues like satiety [35,36]. Such a procedure alleviates maternal discomfort and potentially prolongs the pregnancy, thereby reducing the risk of pPROM and preterm labor, placental abruption, and postpartum hemorrhage due to uterine atony associated with overdistension [34,37]. Amnioreduction strategies are largely subjective and rely solely on US imaging, without clear guidelines regarding the volume and rate of amniotic fluid drainage, or its relationship to amniotic pressure. As a result, there is no universally accepted amnioreduction protocol or defined criteria for terminating the drainage procedure. In the absence of objective measures, the decision to conclude the intervention is typically based on the clinician’s experience and US assessment [38]. The intervention is typically concluded when US examination shows an AFI between 15 and 20 cm, or when intrauterine pressure decreases to below 20 mmHg [33]. A retrospective study conducted by Laoreti A. et al. confirmed the safety of amnioreduction in cases of polyhydramnios in twin pregnancies [34]. The complication rate was low, particularly considering the risks associated with severe polyhydramnios. No cases of chorioamnionitis or sepsis were reported, with only one case of placental abruption and two cases of preterm delivery. The median gestational age at delivery was 36.5 weeks, and the median interval from the first drainage procedure to delivery was 30 days [34]. Another retrospective study also confirmed the low complication rate associated with amnioreduction. However, it did not demonstrate any benefit in terms of pregnancy prolongation [36]. The decision to perform the procedure should be made on an individual basis, taking into account the maternal clinical condition, cervical length, gestational age, and the patient’s preferences. An amnioreduction procedure was proposed in the described case, but the patient refused it. Presumably, this would have prolonged the pregnancy, minimizing the consequences of neonatal prematurity. A controversial aspect of this case is the delivery method. In pregnancies with fetal anomalies, spontaneous vaginal delivery is the primary choice since it is associated with reduced maternal morbidity and mortality. However, cesarean section is indicated if there is a high risk of bleeding, gestational sac rupture, or shoulder dystocia [39]. In this case, the massive polyhydramnios poses a high risk of fetal repositioning during labor. Lastly, the delivery of a fetus with acrania is also classified as technically more difficult. All of the above led to the choice of the cesarean section as the delivery method. While most of the literature focuses on singleton pregnancy cases, reports of acrania in twin pregnancies—particularly when only one fetus is affected—provide unique insights into diagnostic complexities and clinical decision-making. In a case reported by Hata T. et al., a monochorionic diamniotic twin pregnancy was diagnosed with acrania in one fetus between 10 and 13 weeks of gestation [40]. The authors observed unusual early inter-twin contact via three-dimensional ultrasound, noting that the affected fetus frequently approached the healthy co-twin. This case emphasized the need for meticulous early imaging in twin pregnancies, particularly in the setting of suspected structural abnormalities [40]. In a similar context, Thompson and Otigbah presented a case of monochorionic twins discordant for acrania complicated by twin-to-twin transfusion syndrome (TTTS) [41]. Diagnosed at 12 weeks, the acranial fetus exhibited absence of a stomach bubble and impaired swallowing, confounding the assessment of amniotic fluid levels—a key parameter in TTTS staging. Doppler ultrasonography was thus essential for evaluating vascular discordance. Selective laser coagulation and cord occlusion were performed, resulting in the demise of the affected fetus and the survival of the healthy co-twin, delivered preterm at 30 weeks. This case illustrates the added complexity TTTS introduces when coexisting with a lethal anomaly like acrania and the importance of multidisciplinary care [41]. Additionally, a case from Chile involved a 28-year-old woman with a monoamniotic twin pregnancy discordant for acrania, diagnosed at 12 weeks. Ultrasound confirmed the diagnosis and revealed umbilical cord entanglement. Although the parents considered fetoscopic cord occlusion of the affected twin, legal restrictions in Chile prevented this intervention. Consequently, expectant management was pursued, aiming for cesarean delivery at 32 weeks. However, at 25 + 5 weeks, the patient experienced preterm labor, and ultrasound indicated intrauterine demise of the healthy twin. The affected twin was delivered alive but died shortly after birth. Postnatal examination confirmed multiple umbilical cord knots, corroborating prenatal findings. This case underscores the complexities in managing monoamniotic twin pregnancies complicated by lethal anomalies. While selective termination of the affected twin may improve outcomes for the co-twin, ethical and legal considerations can limit available options. Therefore, individualized care plans, incorporating thorough counseling and close monitoring, are essential in such high-risk scenarios [42]. Expanding the phenotypic spectrum, Gibbs, TS. et al. reported a case of exencephaly with ectopia cordis in one fetus of a twin pregnancy—a defect developmentally related to acrania [43]. The combination of these anomalies is exceptionally uncommon and presents significant challenges in prenatal diagnosis and management [43]. A broader analysis was conducted by Kwon, TH. et al., who reviewed 13 cases of acrania, including one in a twin gestation. Their findings reinforced the value of sequential ultrasound [44]. These reports collectively emphasize the importance of early and accurate prenatal diagnosis using high-resolution ultrasonography, supplemented by Doppler studies when TTTS or other complications are suspected. Despite their rarity, twin pregnancies discordant for acrania pose significant clinical and ethical challenges. The lack of standardized management guidelines for such cases highlights the need for further research and the development of evidence-based protocols to improve maternal and neonatal outcomes [40,41,42,43,44].

The prenatal diagnosis of a fetal lethal malformation can have a significant psychological impact on parents, as it is an extremely stressful experience. Typically, parents expect to receive reassuring information about a healthy fetus with normal prenatal development during an ultrasound examination. However, any diagnosis of a fetal anomaly alters their expectations regarding the course of the pregnancy [45]. Carlsson, T. et al. proposed a three-phase model of the psychological response following the diagnosis of a fetal anomaly, which can be described as an emotional rollercoaster [46]. The first phase is shock, representing an unexpected and deeply personal tragedy for the parents. The information is difficult to comprehend and forces them to make a challenging decision about the future—whether to terminate or continue the pregnancy. The next phase is described as an existential crisis. As the initial shock fades, parents may experience a profound emotional struggle. This phase often includes feelings of guilt, as they may question their role in the development of the fetal anomaly, along with emotions such as unfairness, anger, and helplessness. Insomnia is also common during this period. The final phase is life remodeling, in which parents come to terms with the situation and begin to prepare for the birth. Throughout all stages, parents are burdened with concerns about the course of the pregnancy and the postnatal outcome [46,47]. Detecting fetal anomalies can lead to psychiatric disorders such as anxiety, depression, and post-traumatic stress disorder in both parents. Oftedal, A. et al. analyzed the impact of prenatal diagnosis of congenital anomalies on parental mental health compared to parents with uncomplicated pregnancies [10]. Mothers receiving such diagnoses had significantly higher levels of depressive symptoms (χ²(1) = 73.89, *p* < 0.001, mean difference = 4.46 ± 0.47; Edinburgh Postnatal Depression Scale). Fathers also showed elevated scores (χ²(1) = 40.75, *p* < 0.001, mean difference = 2.80 ± 0.42). Traumatic stress, measured using the Impact of Event Scale, was greater among parents of fetuses with diagnosed anomalies. The severity of the anomaly did not affect fathers’ levels of depression or stress, but it was associated with increased maternal intrusion and avoidance (χ²(1) = 3.90, *p* < 0.01, mean difference = 4.72 ± 2.39; χ²(1) = 2.15, *p* < 0.05, mean difference = 2.79 ± 1.91) [10]. A multidisciplinary approach to managing such pregnancies is essential and should include psychological or psychiatric support for both parents. This psychological burden becomes even more complex in the context of twin pregnancies, which are associated with an elevated risk of psychiatric disorders [48]. The diagnosis of a lethal anomaly in one of the fetuses may lead to increased pregnancy-related stress, anxiety, depression, and, in severe cases, even tokophobia. Preterm labor, preterm prelabor rupture of the membrane, and low birth weight are just some of the possible consequences of increasing hypothalamic–pituitary–adrenal activity [49,50]. Additionally, the fetal exposition on stress hormones during pregnancy may have a lifespan consequence, including neurodevelopmental disorders, such as attention, cognitive, or motor dysfunctions. Also, it can result in long-term issues such as emotional, temper, or behavioral problems in adolescents [50]. Noteworthy, the high level of cortisol has an impact on fetal immune system development, which may cause asthma or severe allergies. It is possibly related to the decreasing number of immune cells in the fetal thymus and spleen due to stress hormone activity. On the other hand, maternal adverse pregnancy outcomes such as preeclampsia, or gestational diabetes can also be caused by high stress levels [49]. That is why psychological or psychiatric support may be a significant part of antenatal care of pregnant women in similar cases. Moreover, in particular circumstances, a place in a dedicated perinatal hospice with a multidisciplinary medical team should be offered [51].

## 4. Conclusions

Acrania is a rare and lethal neural tube defect, resulting from early embryological disruption in cranial development. Understanding its pathogenesis, as well as its potential genetic associations, is essential in the context of prenatal diagnostics and counseling. High-resolution US and fetal MRI remain critical tools in identifying structural abnormalities during the first trimester. This case highlights that with individualized and holistic care, outpatient monitoring may be feasible in twin pregnancies complicated by a lethal anomaly in one fetus. Psychological support and ethical considerations are essential components of care in such cases. Finally, preventive strategies remain crucial—regular periconceptional folic acid supplementation should be strongly promoted, as it significantly reduces the risk of neural tube defects. Further research should continue to refine genetic counseling approaches and evaluate both clinical outcomes and long-term psychosocial impact in similar high-risk pregnancies.

## Data Availability

The original data presented in this study are available on reasonable request from the corresponding author. The data are not publicly available due to ethical restrictions.

## Abbreviations

The following abbreviations are used in this manuscript:

AEAS	Acrania–exencephaly–anencephaly sequence
NTDs	Neural tube defects
US	Ultrasound
MRI	Magnetic resonance imaging
pPROM	Premature preterm rupture of membrane
MVP	Maximal volume pocket
AFI	Amniotic fluid index
TTTS	Twin-to-twin transfusion syndrome
MTHFR	Methylenetetrahydofolate reductase gene
VANGL1	Van Gogh-like planar cell polarity protein 1
